# Visual programming for next-generation sequencing data analytics

**DOI:** 10.1186/s13040-016-0095-3

**Published:** 2016-04-27

**Authors:** Franco Milicchio, Rebecca Rose, Jiang Bian, Jae Min, Mattia Prosperi

**Affiliations:** Department of Engineering, Roma Tre University, Rome, Italy; Bioinfoexperts, LLC, Thibodaux, LA USA; Department of Health Outcomes and Policy, University of Florida, Gainesville, FL USA; Department of Epidemiology, College of Public Health and Health Professions & College of Medicine, University of Florida, 2004 Mowry Road, Gainesville, 32610-0231 FL USA

**Keywords:** Next-generation sequencing, High-throughput sequencing, Big data, Template library, Generic programming, Visual programming, Graphical user interface, Software suite

## Abstract

**Background:**

High-throughput or next-generation sequencing (NGS) technologies have become an established and affordable experimental framework in biological and medical sciences for all basic and translational research. Processing and analyzing NGS data is challenging. NGS data are big, heterogeneous, sparse, and error prone. Although a plethora of tools for NGS data analysis has emerged in the past decade, (i) software development is still lagging behind data generation capabilities, and (ii) there is a ‘cultural’ gap between the end user and the developer.

**Text:**

Generic software template libraries specifically developed for NGS can help in dealing with the former problem, whilst coupling template libraries with visual programming may help with the latter. Here we scrutinize the state-of-the-art low-level software libraries implemented specifically for NGS and graphical tools for NGS analytics. An ideal developing environment for NGS should be modular (with a native library interface), scalable in computational methods (i.e. serial, multithread, distributed), transparent (platform-independent), interoperable (with external software interface), and usable (via an intuitive graphical user interface). These characteristics should facilitate both the run of standardized NGS pipelines and the development of new workflows based on technological advancements or users’ needs. We discuss in detail the potential of a computational framework blending generic template programming and visual programming that addresses all of the current limitations.

**Conclusion:**

In the long term, a proper, well-developed (although not necessarily unique) software framework will bridge the current gap between data generation and hypothesis testing. This will eventually facilitate the development of novel diagnostic tools embedded in routine healthcare.

## Main text

### Background

High-throughput or next-generation sequencing (NGS) technologies have become an established and affordable experimental framework for basic and translational research in biomedical sciences and clinical diagnostics [1–3]. The applications of NGS are almost endless, spanning many ‘–omics’ fields, such as genomics, transcriptomics, and metabolomics [3–11]. Nowadays, it is possible to sequence any microbial organism or metagenomic sample within hours and to obtain human genomes in weeks. By sequencing the entire genome in targeted patients, it is possible to identify genes and regulatory elements related to pathophysiological conditions. Genome-wide association studies and analysis of gene expression, usually made via well-established microarray techniques, can now be done via NGS, e.g. RNA-Seq[uencing]. NGS allows for full genome characterization of other organisms besides the human genome, including known pathogens, and yet-to-be-identified bacterial, viral, or fungal species that may pose a public health threat [12]. Another growing application of NGS is microbial community analysis. The diverse host-associated microbiota has received intense research interests for its potential associations with human health outcomes [13]. With few modifications in sample preparation protocols, a single NGS machine can offer the scientist an abundance of data for exploring multi-domain research questions.

Several NGS platforms and sequencing technologies are available [14]. Technology providers include Illumina Inc. [15], Thermo Fisher Scientific [16], Roche [17], and Pacific Biosciences [18]. NGS services are available at a comparable price to established sequencing methods such as Sanger, although with considerably greater data output [19–21].

Whereas the traditional Sanger [22] approach produces contiguous nucleotide sequence reads between 400 and 700 bases with a throughput of 50-30,000 kilobases per hour, NGS reaches a throughput of 10-600 gigabases per hour, producing reads up to 700 nucleotide bases long [9], and Pacific Biosciences broke the 10,000+ bases length record. The terabyte-size of nucleotide sequence data per run is becoming a reality, which will further lower per-sample sequencing cost [23, 24]. The fourth-generation of Oxford’s Nanopore-based sequencers have the potential to reduce the cost for sequencing an entire human genome from the fairly recent $1,000 target [25] to an astounding $100 [26, 27]. The decreasing trend of the cost-per-base of DNA sequence since 2008 even exceeded Moore’s law [28], i.e. the exponential growth of computing hardware capabilities, where the number of transistors in an integrated circuit doubles approximately every two years.

Ever since the first NGS machine was commercialized in 2004 by 454, the development of robust, intuitive, and easy to use analytic tools has been behind data generation capabilities. This state was defined with the evocative term “analysis paralysis” in 2010 [29]. A landmark paper in 2012 by Vyverman et al. highlighted the limitations and needs of bioinformatics tools for a variety of complex string problems that are at the base of most NGS analytics [30]. Five years later, analysis is no longer paralyzed. A plethora of NGS data analysis software has emerged, with considerable redundancy. Nevertheless, software development must adapt to handle fast-pace evolving technology, e.g. further data inflation resulting from the Nanopore platform [31–34].

Most of the current NGS software requires dedicated bioinformaticians with access to comprehensive computational infrastructure. Just a few years ago, there was a bottleneck between data generation and inference (analyzing and making sense of the data), but nowadays, access to these bioinformatics resources is more common and affordable. The new bottleneck is the evolution of software in accordance with technological advances and users’ needs.

Comprehensive software suites for NGS analytics must be supported by an appropriate development environment. The lack of an organized programming base slows down the development of innovative applications that can be handled directly by the investigators generating the data. Biological scientists carrying out experiments at times undergo delays and difficulties in analyzing NGS data because tools customized to their needs and abilities are not readily available. Current software for NGS analytics requires medium-to-advanced level of computational proficiency. One reason is the compulsory use of high-performance computing infrastructure for analyzing most NGS data sets. Such computational arrangements should not be necessary when sequencing individual fungal, microbial or viral pathogens or when performing targeted phylogenetic studies (e.g. 16S ribosomal RNA); a desktop computer should be sufficient for analyzing bacterial data generated by platforms such as Illumina’s MiSeq. When users need to move onto a high-performance computing infrastructure for projects involving large numbers of human genome sequences, they may benefit from the availability of software they are already familiar with (i.e. the one running on their desktop machine), rather than being required to learn an entirely new set of programs. An example in statistical analytics is the SAS software system (SAS Institute Inc.), which does not require the users to change the programming syntax when migrating across different components or installations (including desktop, server, and distributed editions).

At present, software engineers who develop new algorithms and analytical tools for NGS face a lack of dedicated libraries and interoperable software, and they have to write new tools which in turn cannot be interoperable. From a developer’s perspective, many existing programs could be rewritten to be more efficient or to be parallelized homogeneously, as in hierarchical build of programs, for easy integration across various platforms. With a common software layer that abstracts interactions between data and algorithms, integrating procedures that exploit multithreading or distributed computing may be achieved without in-depth modifications of the algorithms themselves. In addition, the adoption of generic programming template libraries can homogenize programmers’ work and permit a more community-engaged software development.

### Template libraries and generic programming

In spite of the glut of NGS software [35], there is a lack of low-level programming approaches; in other words, the development of specific data structures and functions (e.g. a de Bruijn graph constructor or a Burrows-Wheeler transformation function) for languages like C++ or Java are in short supply. Software packages and libraries specifically designed for NGS such as BAMTools [36], htslib (SAMtools/bcftools) [37], NGS++ [38], Bioclojure [39], or libStatGen [40] are focused on parsing and file format standardization, with limited provision of data structures and algorithms useful for NGS analytics. Although a number of libraries and toolsets for generic sequence analysis is available [41–43], their incorporation into NGS generic programming is problematic given the tremendous shift in data size. This is also true for programming language extensions such as BioPerl, BioRuby, BioJava, BioPython [44–47], born under the unifying effort of the Open Bioinformatics Foundation [48] and for large repositories like Bioconductor [49, 50]. Note that we differentiate between true programming libraries, toolkits, and software tools [51]. A library is a collection of data structures and functions/methods for a specific programming language (usually written in the same language, but not necessarily if the language is at a high-level, like R), which can be used seamlessly when writing new code in that language. A toolkit deviates from the rigorous concept of library as it can also include a set of executable programs which can be called and combined internally or externally (like EMBOSS). Lastly, a software tool is a standalone program that has a fixed input/output routine and whose internal functions or data structures cannot be used elsewhere. For instance, the popular BWA program for mapping short reads to a reference is a standalone program, even if it features internal data structures like the Burrows-Wheeler transform, used by other programs, like Bowtie. Table 1 gives a description of the most popular libraries and toolsets for sequence analysis and NGS data processing. One example of a sequence analysis library that evolved successfully to handle NGS data is SeqAn [52, 53]. This was possible because according to SeqAn website: “SeqAn applies a unique generic design that guarantees high performance, generality, extensibility, and integration with other libraries.” The SeqAn library is written in C++ and licensed as an open source. It also employs the Hierarchical Data Format 5, which makes possible the management of large and complex data collections [54] in serial, multithreaded, and distributed environments. The number of tools for NGS that have been released using SeqAn is remarkable and proves how such open programming approach is advantageous [55–57]. Bowtie, Lambda and Fiona are written in SeqAn, the latter of which is one of the fastest local aligners for NGS data and error correction tool, and it may become an alternative to BLAST. Another toolset similar to SeqAn is GenomeTools [58], which is efficient but provides limited functionality and genericity. To our knowledge, SeqAn is the only available NGS-specific library that embraces the generic programming philosophy.

**Table 1:** Summary of programming libraries/toolkits for analysis of (next-generation) sequencing data

Library Name	Release Date	Programming Language	License	Website	Features
EMBOSS [43]	2000	C C++ BTL others	GNU GPL	http://emboss.sourceforge.net/	Sequence alignment; rapid database search; protein motif identification; nucleotide sequence pattern analysis; codon usage analysis for small genomes; rapid identification of sequence patterns in large scale sequence sets; presentation tools for publication.
BTL [41]	2001	C++	GNU GPL	http://www.cryst.bbk.ac.uk/~classlib/	Data structures (e.g. graphs); nucleotide string methods (e.g. Fourier transform, Needleman-Wunsch alignment).
Bioperl [47]	2002	Perl	Artistic License GNU GPL	http://bioperl.org/	Access sequence data from local/remote data bases; manage data base formats; data base search; manipulating sequences/sequence alignments; gene annotations.
Bioconductor [50]	2003	R (C/C++)	Artistic BSD GNU GPL	https://www.bioconductor.org/	Repository of multiple libraries for analysis and comprehension of genomic and –omics data, including NGS.
BioPHP	2003	PHP	GNU GPL	http://biophp.org/	DNA and protein sequence analysis, sequence alignment.
GenomeTools [58]	2003	C	Open BSD	http://genometools.org/	Parsing, compression, k-mer, suffix trees, annotation, error correction and other sequence analytics (FASTA, FASTQ)
Pizza&Chili [94]	2005	C/C++	GNU Lesser GPL	http://pizzachili.di.unipi.it/	Compressed indices, text collections
Bio++[42]	2006	C++	CeCILL GPL	http://kimura.univ-montp2.fr/BioPP	Sequence analysis, phylogenetics, molecular evolution; population genetics.
Biojava [46]	2008	Java	GNU Lesser GPL	www.biojava.org/	Manipulate biological sequences; file parse; DAS client/server support; access to BioSQL/Ensembl data bases; tools for making sequence analysis GUIs; statistical routines; dynamic programming toolkit.
SeqAn [52]	2008	C++	BSD 3-clause	http://www.seqan.de/	Extensive set of algorithms and data structures for the analysis of nucleotide sequences, with emphasis on NGS data; includes index, compression, data base search, support for NGS-specific file formats (fastq, SAM/BAM, VCF, BED).
Biopython [45]	2009	Python, C	Biopython	http://biopython.org/	Sequence input/output; alignment input/output; population genetics; structural bioinformatics; SQL interface.
htslib SAMtools BCFtools [37]	2009	C	MIT Expat Modified BSD	http://www.htslib.org/	Read, write, edit, index, view SAM/BAM/CRAM formats; read, write BCF2/VCF/gVCF files; call, filter, summarize SNP/short indels.
BioRuby [44]	2010	Ruby	GNU GPL	http://bioruby.open-bio.org/	DNA and protein sequence analysis, sequence alignment, biological database parsing, ontology, structural biology.
BAMTools [36]	2011	C++	MIT	https://github.com/pezmaster31/bamtools	Read, write, manipulate BAM formats
libStatGen [40]	2011	C++	GNU GPL	https://github.com/statgen/libStatGen	Handle SAM/BAM, fastq, GLF, VCF, ASP.
NGS++ [38]	2013	C++	GNU Lesser GPL	https://github.com/NGS-lib/NGSplusplus	Read, write, manipulate multiple genomic file formats and data associated with BED type files (epigenomics).
Bioclojure [39]	2014	Clojure	GNU Lesser GPL	https://github.com/s312569/clj-biosequence	Parse of Genbank, Uniprot XML, fasta, fastq formats; wrappers for BLAST, signalP, TMHMM; index files for random access, lazy processing of sequences from very large files.

### What is template generic programming?

A generic programming framework provides *traits classes* [59], i.e. an abstract representation of data types and algorithms [60]. As a real-life example, take the LEGO® toy construction kits. A child (end user) wants a LEGO® model of a brick that resists trampling. A company (developer) may be contracted to produce new stomp-resistant brick types (data structures/functions) that respect the general specification (traits class) of LEGO® bricks (e.g. hole spacing and size), thus ensuring that, before reaching the end-user, the new brick (structure) will work with any other LEGO® toy piece. In essence, a traits class may be seen as a prescription, a specification for data structures or functions: it enforces clients to respect a list of prerequisites. More technically, a traits class is the gateway for calling a function on a generic and a priori unknown data structure, employed at compile time (that is parametric polymorphism). If the given data structure provides types and methods definitions required by the traits, then any algorithm employing such a structure may be applied to any data structure that respects the requirements. This allows developers to write algorithms that can be applied at no runtime cost to any data structure, without knowing a priori which data structure will be employed or the types involved in the process.

Template generic programming enables provision of seamless infrastructure for computational tasks and replacement of serial algorithms, or data types with multithreaded/distributed ones, without significant changes to the program structure and without additional runtime overhead, provided the adherence to a traits class. For instance, when reading molecular sequence files and associated metadata (e.g. fastq files with nucleotides and quality scores), the compiler generates an ad hoc class given a chosen structure (e.g. a list or a suffix tree); no virtual functions and inheritance will be involved, reducing the runtime overhead. One could replace a serial constructor with a distributed one (e.g. reading sequence files in parallel), or change its associated methods (e.g. a sequence corrector based on two different algorithms) without changing the program; since the software employs traits to access a generic data structure, the compiler will ensure that the change will be a valid one. Hence, when a container does not respect the requirements, the software will refuse to compile, allowing developers to assess the interoperability of data types and algorithms without any performance degradation, and to write software libraries that may be applied to any traits-abiding data structure. The advantage of a library on generic programming templates is straightforward: the library can be updated for handling new data types, increase in data size, and technology changes in computing, like a new graphic processing unit chip or a computation accelerator (e.g. Intel® Xeon Phi).

### General-purpose software suites and graphical user interfaces

Low-level libraries and command-line toolsets are appropriate for software developers and programming-savvy users; they form the base to develop high-level ensemble software suites that feature a graphical user interface (GUI) with menus and premade workflows or analysis pipelines. Suites bring complex analytical procedures to the general user. For instance, user-friendly statistical software with powerful GUI includes Statistica (Dell Inc.), SAS Enterprise (SAS Institute Inc.), and SPSS (IBM Corporation). This easily accessible interface is advantageous as it can facilitate the applied and translational aspects of NGS research for those without programming knowledge, but the software must be carefully designed in order to prevent users from making errors due to lack of specialized expertise. In addition to a GUI featuring single functions and premade workflows, some high-level suites offer visual workflow builders. Visual builders are different form GUIs because they permit combination (more or less flexibly) of existing program functions into new data processing pipelines, which may not be available as pull-down menus or icons in a GUI.

Several high-level tools for NGS analytics are available (see Table 2 for a summary). There are commercial products with closed source code, such as Illumina’s BaseSpace [61], CLCBio [62], DNASTAR’s Lasergene [63], and Geneious [64]. Free and hybrid commercial/free software include Galaxy [65, 66], Globus Genomics [67], PATRIC [68], and UGENE [69, 70]. Most of the software suites can be installed locally or on a server.

**Table 2:** Summary of all-purpose software suites for analysis of next-generation sequencing data offered with a graphical user interface option

Software Name	License	Free	Platform	Installation	Workflow Builder	Website
BaseSpace	Proprietary	No	Web-browser App	Cloud	No	https://basespace.illumina.com
CLCBio	Proprietary	Trial	Web-browser	Server	Yes	http://www.clcbio.com/
DNASTAR Lasergene	Proprietary	Trial	MS Windows Mac OSX UNIX/Linux	Localhost Server	No	http://www.dnastar.com/
Galaxy	GNU GPL	Yes	Web-browser	Localhost Server Cloud	Yes	https://galaxyproject.org/ https://usegalaxy.org/
Geneious	Proprietary	Trial	MS Windows Mac OSX UNIX/Linux	Localhost Server	No	http://www.geneious.com/
Globus Genomics	Apache + third party	Yes/No (depends on the service)	Web-browser	Cloud	Yes	https://www.globus.org/genomics
Golden Helix	Proprietary	Trial	MS Windows Mac OSX UNIX/Linux	Localhost	No	http://goldenhelix.com/
Partek	Proprietary	Trial	MS Windows Mac OSX UNIX/Linux	Localhost	No	http://www.partek.com/
PATRIC	GNU GPL	Yes	Web-browser	Cloud	No	https://www.patricbrc.org
Sequencher	Proprietary	Trial	MS Windows Mac OSX	Localhost Server	No	https://www.genecodes.com/
SevenBridges	GNU GPL (Rabix) + third party	Trial	Web-browser	Cloud	Yes	https://www.sbgenomics.com/
SoftGenetics	Proprietary	Trial	MS Windows Mac OSX (via Parallels)	Localhost Server	No	http://www.softgenetics.com/
UGENE	GNU GPL	Yes	MS Windows Mac OSX UNIX/Linux	Localhost Server	Yes	http://ugene.net/
Vector NTI	Proprietary	Trial	MS Windows Mac OSX	Localhost Server	No	http://www.thermofisher.com/us/en/home/life-science/cloning/vector-nti-software.html

Illumina’s BaseSpace provides an exclusively cloud-based environment hosted by Amazon Inc., direct integration with sequencing instruments, and workflows as mobile-touch apps.

Galaxy, a web-browser application, is one of the most popular, open source NGS suites and effectively offers a unique developer’s platform, permitting the integration and collation of different programs. Galaxy has a substantial support from the developers’ community; both researchers and federal agencies are investing in this promising platform, which also features a visual workflow builder. However, Galaxy does not permit development of new algorithms and standalone software by itself, and it must be supported by a proper collection of low-level programs.

UGENE is another free and open source platform. The suite is multiplatform, i.e. it runs on computers with any operating system, such as MS Windows or Mac OSX by using the Qt C++ framework, incorporates multiple pipelines for NGS, and allows visual design of new ones with its built-in workflow builder, as in Galaxy (with similar limitations).

Not all procedures implementable from the command-line can be represented in the Galaxy’s workflow builder. For instance, workflows require a fixed set of inputs and therefore looping on files within is not yet available (as of November 2015). ‘Enhanced’ Galaxy frameworks like Globus Genomics or SevenBridges can overcome such workflow limitations, but require a higher technical expertise [67]. UGENE’s builder has limitations similar to Galaxy. New features in UGENE can be requested to the developers’ team, which also provides tech and other types of support for a price. UGENE is equipped for parallel computation, but the capabilities to distribute jobs are limited by the capabilities of programs embedded (also the case for Galaxy). Nonetheless, UGENE developers’ team has steadily published new workflows for NGS, providing extensive walkthroughs for the non-specialists: recently released pipelines include: “Variant Calling with SAMtools,” “Tuxedo Pipeline for RNA-seq Data Analysis,” and “Cistrome Pipeline for ChIP-seq Data Analysis,” all currently integrated into the Unipro UGENE desktop toolkit [69].

### Visual programming

We have highlighted the importance of creating a solid low-level base for NGS programming and a high-level base to scale up analytics, especially with the usage of visual tools.

In computer science, a visual programming (VP) language is a *medium* for implementing computer programs that makes uses of graphical operators and elements rather than textual ones. VP is not a new concept [71–74]; it has been envisioned in several ways starting from the early 1960s and has been the object of philosophical debates [75, 76]. VP is different from GUI. A GUI aids users executing programs via visual menu items in contrast to command-line (i.e. terminal) text scripting. In general, GUI menus are premade and users cannot create new programs or combine menu functions within the GUI. Conversely, a VP language has the same power as a textual programming language or a library, if it features the same functional elements (e.g. data structures and methods); therefore, new algorithms and programs can be designed and compiled within a VP, and VP can even be used to implement GUIs. Visual approaches to programming have been explored in diverse environments, including education, multimedia, system simulation and automation, data warehousing, and business intelligence, with probably the most successful example being the computer-aided design (CAD) software industry. Another extremely popular area for VP is video game design [77, 78]. Although in principle VP can be used to create algorithms starting from the lowest hierarchy of programming language elements, in practice, VP is employed for creating higher-level applications using libraries. This facilitates developers’ work when a large amount of coding (and redundant coding) is required.

### How can visual programming benefit NGS software development?

Currently, there are no ‘pure’ VP approaches being developed for NGS applications. Galaxy or UGENE workflow builders can be considered rudimental VP environments, but as discussed previously, they do not offer the same set of functions as the command-line and have limited interoperability (i.e. they work only within their parent environment and cannot build independent programs). However, there is potential for improving the workflow builders using the VP approach.

### Visual programming entities

The main elements of a VP language are: *building blocks*, *block engines*, *block connectors*, and *meta-blocks.* Building blocks are the basic VP pieces, like LEGO®; they can have different functions, like the different shapes of LEGO® blocks. Building blocks can represent a file parser, a read trimmer, a mapping algorithm, a *k*-mer graph builder, a SAM/BAM file converter, et cetera. They can be data structures, constructors, methods. Technically, building blocks are filters modelled functionally, linking connectors with input-output control, in accordance to the domain-driven design paradigm, e.g. C# or Microsoft.NET [79, 80]. Block engines perform computational procedures within a building block; for example, they read a fastq file or they map a read set to a reference genome using a specific algorithm. In the generic template framework, the algorithms can be transparently replaced (given that implementations respect devised traits classes). Block connectors are relational mappings between data structures and algorithms within functional visual blocks, i.e. the communication links among building blocks. For instance, a read mapper requires both a reference sequence (which could be indexed upon parsing) and a read set (which could be read as single- or paired-end). A metagenomics classifier requires a genomic database and the read sets. Block connectors encode these relations. Finally, meta-blocks are blocks made of multiple building blocks and connectors, i.e. whole data analysis pipelines, replaced by a single building block of a higher hierarchy (given a defined ontology for the blocks). If a user is not a developer, there is no need for them to access the whole block workflow. They can use the blocks at the highest level hierarchy which would correspond to a single icon/menu in the GUI; for instance, a “metagenomics analysis pipeline” meta-block would ask for a specific set of input files (fastq) and write a standardized output (fasta, KEGG, SEED). The more a user is acquainted with NGS algorithms, the more they can get trained into the visual programming interface, without resorting to traditional text-based programming. This is a convenient trade-off between black-box and informed design/use.

**Fig. 1:**
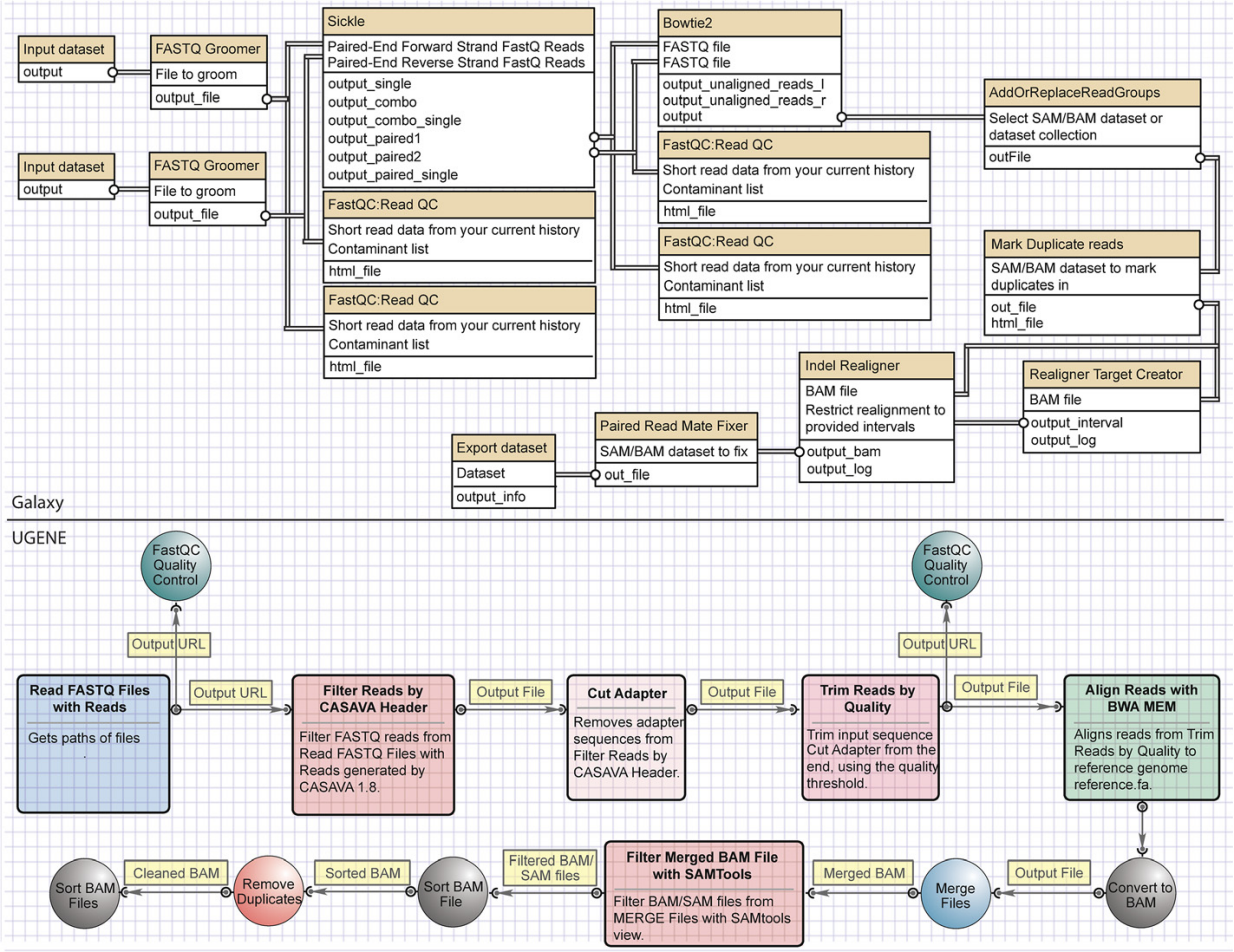
Example of a pipeline for single nucleotide variant calling from fastq files, using Galaxy’s (top) and UGENE’s (bottom) workflow builders

Figure 1 shows an example of pipelines for single nucleotide variant calling from fastq files, using UGENE’s and Galaxy’s workflow builders. Both workflow builders feature block connectors that well represent the aforementioned VP entity. Likewise, one could consider a saved workflow as a meta-block. Building blocks and block engines are inherently fused, and each block corresponds to a program that has been installed in Galaxy or UGENE (e.g. Sickle, CASAVA, Bowtie2, BWA). If any of the workflow could allow access functions of a generic programming library (e.g. SeqAn), with a corresponding distinction between block hierarchies and functionality, there would be an improvement in expressive power, which would not be limited to the sole ability of pipelining compiled programs. Notably, the UGENE workflow builder is made with the cross-platform Qt C++ Library, which is an ideal development framework for enabling such transition.

### Physiognomy of visual programming

**Fig. 2:**
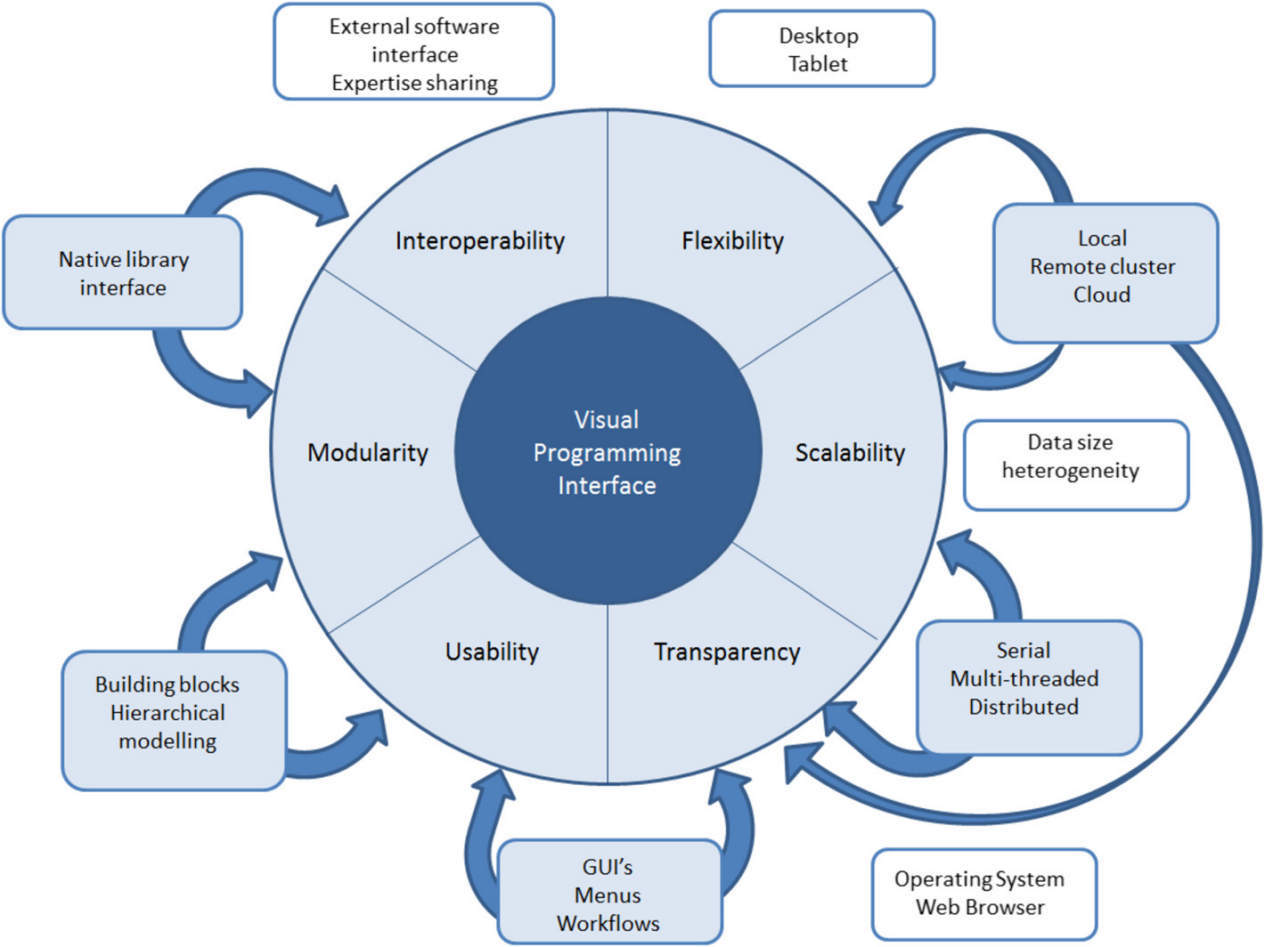
Physiognomy of visual programming for development of tools for next-generation sequencing data analytics

By definition, VP facilitates various aspects of the process of software design and brings closer the user and the developer. Development policies for VP should follow the same prerogatives. One way to achieve this can be through the adoption of the Agile methodology [81], which implements code and develops user interfaces at the same time, with an adaptive strategy of real-time planning. Agile is an efficient and face-to-face communication between developers, stakeholders, and final users, blending the characteristics of different disciplines directly into the final product. We believe the Agile method can be appropriate for development of NGS tools via VP, when a library base is already available (e.g. SeqAn), yet it must be delivered to users with a different expertise and must meet their usage needs. Independently of the chosen development methodology, a VP framework for NGS should meet the following requirements: *flexibility*, *scalability*, *transparency*, *usability*, *modularity*, and *interoperability*. As summarized in Fig. 2, each of these characteristics affects either a user’s or a developer’s needs. For instance, flexibility of a VP product concerns its capacity to be workable on different devices such as desktops vs. tablets, or feasible for local/server/cloud installations (which is different from being installable on different operating systems or being usable). Scalability refers to the capacity of the software to scale up with data size increase, by featuring different solutions, such as switching to multithreaded mode or moving analyses from a localhost to the cloud. Note that there are needs assessments falling in multiple categories. In fact, the dichotomy of multithreading (say, parallelization within graphic processing units or central processing units) vs. distribution (e.g. message parsing interface) do relate to scalability, but also to transparency, especially in the case of adoption of a generic template library programming paradigm. As already mentioned, transparency can be associated with multiplatform (any operating system) or web-browser-based implementations. The VP framework and its products must be transparent not only at the operating system level but also at the hosting level (i.e. local host vs. cloud). In particular, security on cloud-based computations plays a major role in the potential applicability of NGS software. In regards to usability, we have already mentioned the potential advantage of employing the user-oriented Agile methodology. Any product developed using VP has to be subject to the same verification, validation, quality assurance standards, and common GUI testing schemes [82] like other software, e.g. the Institute of Electrical and Electronics Engineers (IEEE) standard verification and validation IEEE 1012 or quality assurance IEEE 730 [83]. Within a developer’s VP environment, usability can mean a well-designed set of building blocks and functions. These are also related to modularity, where the availability of a native generic template programming library can make a difference. Finally, interoperability can be divided into three levels. The first one is software interoperability, which ensures the possibility of using external pieces of software or libraries. The second level is semantic interoperability, which is the ability to exchange data with unambiguous, shared meaning; when developing NGS tools this involves keeping track of meta-data information such as library versioning, file formats, and file interchange formats. The third level is expertise interoperability, connecting the stakeholders together efficiently by using an appropriate communication infrastructure, such as a users’ forum, like SEQanswers [84], or a developers’ space, GitHub [85].

As an example, Galaxy has strengths in expertise interoperability (thanks to a nourished developers’ and users’ community), software interoperability (by incorporating a plethora of different software and toolsets), and transparency (through the web-browser interface). There is relevant, yet limited, modularity (graphic workflow builder), and scalability (tool parallelization and server installation). Flexibility is enabled by the web-browser GUI. Scalability and interoperability, however, are also subject to original tools’ capabilities and may not keep up with the pace of the current technology evolution. Recent efforts in interoperability for development and standardization of NGS pipelines (i.e. importable in Galaxy and other frameworks) have been concretized in the open source Rabix toolkit [86] for developing and running portable workflows based on the Common Workflow Language specification [87]. Illumina’s BaseSpace and UGENE are strong in usability and flexibility, but are behind Galaxy in other requisites; none of the available suites has a proper VP interface.

**Fig. 3:**
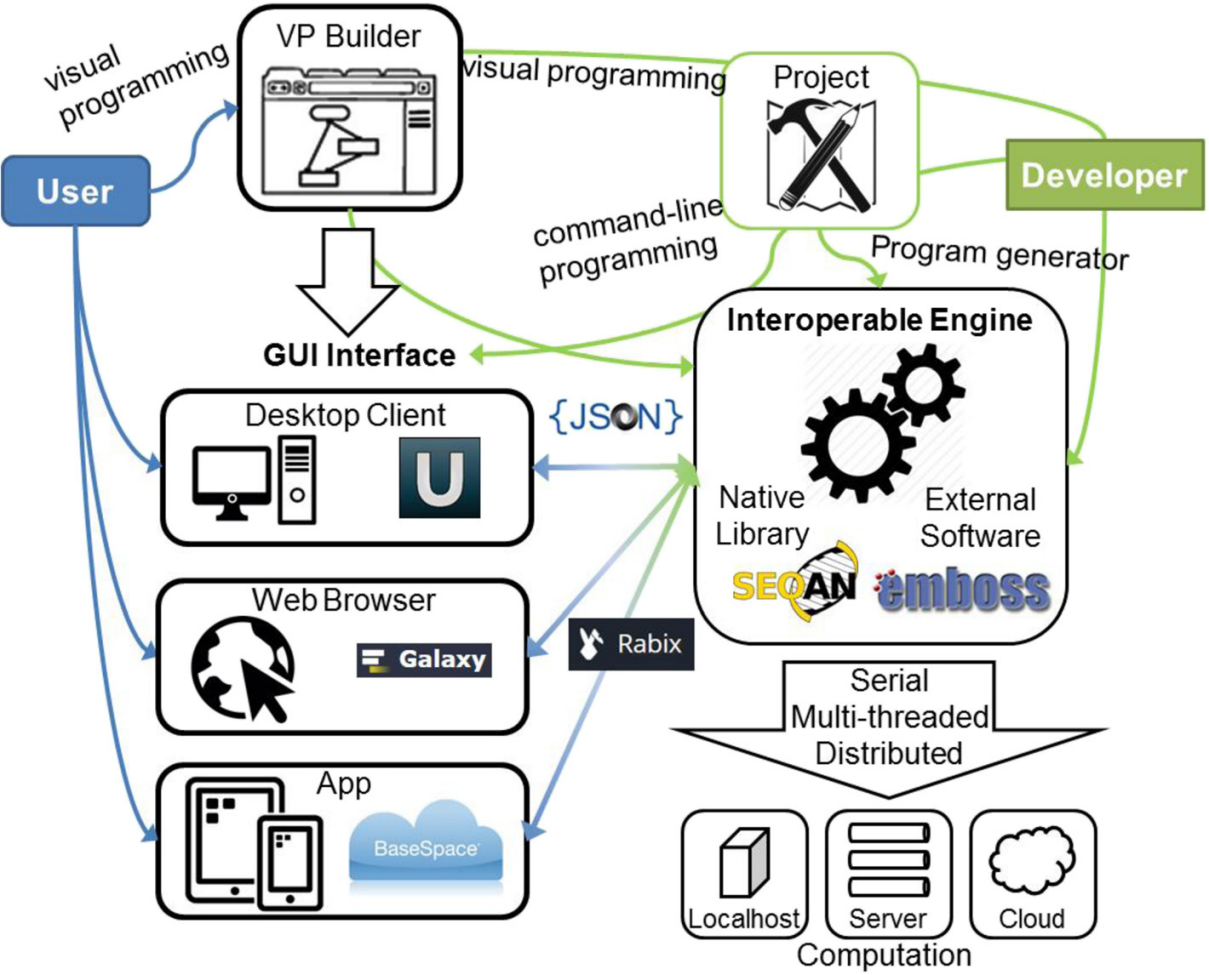
The conceptual visual programming (VP) framework for developing next-generation sequencing data analytics tools

A conceptual VP framework that incorporates many of the prerogatives, named VisPro, has been proposed by Milicchio et al. in 2005 [88], with a case study tailored to development of tools for complex geometric routines. VisPro was developed using the cross-platform Qt Library, like UGENE. More recently, VisPro was extended to web-based applications securely connected to the cloud [89]. In Fig. 3, a diagram of the VP framework for developing next-generation sequencing analytics tools is shown. The VP *Builder* is the graphical development unit, i.e. the VP environment, where both an end user (blue) and a developer (green) can design new program workflows. The end user has access to the *App* and/or the *Web Browser* (i.e. different GUIs) with predefined pipelines which can be made with the VP Builder or even with command-line programming (e.g. a VP *Project*). The developer has access to all the low-level, command-line features besides the VP. The VP *Interoperable Engine* contains all the functions of a generic programming template library, provides compatibility with external programs, e.g. with the JavaScript Object Notation [90, 91] as with Rabix, and runs locally or in the cloud. VisPro prefigured a secure solution based on a client-server architecture for scheduling programs in the cloud [89]; the computational kernel that actually executes a program may be local or remote (i.e. on a cluster), and in the latter case, the client submits, via an encrypted channel and with a secure authentication method –such as secure-socket layer (SSL) certificates or Kerberos [92, 93]– the visual program to a server. The remote scheduler then executes the program on all available nodes as resources become available, leaving the client free to perform other operations. Selecting a local or remote kernel would require only a minimal user intervention, i.e. login host, user name, and password.

### Discussion

NGS data science (or analytics) is an interdisciplinary and critical field of bioinformatics research that has gained increased attention and visibility upon the explosion of NGS technology. It is a sector which has to keep up with the tremendous advancement in sequencing yield and ‘next-NGS’ (future generation) technologies. NGS data science is feasible when proper software is available and can routinely and reliably be used, with reasonable resource spending. The development of software tools for NGS analytics is challenging, given multiple practical hurdles that include the large data sizes, data heterogeneity, and data errors. Despite the glut of NGS software released in the past years, the toolsets are not yet homogenized as they are in other fields (e.g. statistics, automation). We have reviewed the current panorama of low-level software for NGS (i.e. libraries and toolsets) used mainly by developers, and high-level suites (i.e. all-purpose programs with GUIs) used by stakeholders, biological scientists performing experiments for instance. Among the reviewed software libraries, we have identified a positive effort of the Open Bioinformatics Foundation in promoting the ‘Bio’ extensions to programming languages, such as BioJava, BioPerl, BioRuby, BioPHP, et cetera. However, these toolsets are often not well calibrated for the NGS needs (e.g. scalability of methods). Among the NGS-specific libraries, we have identified SeqAn (open source, C++) as the most promising one, because on top of its specificity, it is a generic programming template framework that can seamlessly upgrade itself. SeqAn has already been used in many proof-of-concept works providing efficient/optimized re-implementation of existing methods. Still, low-level libraries are instruments for software developers, not for end users. For the latter, high-level software suites with user-friendly GUIs are available. We have reviewed both commercial and free suites, including Galaxy and Geneious. This general-purpose software usually wraps around existing command-line tools, which may not have been programmed using consistent libraries or programming languages. The suites offer premade pipelines to analyze specific data sets (e.g. RNASeq) with a simple click, combining different programs together. Usability should be the key feature of these graphical suites. Some of the suites also offer the possibility to create ad hoc workflows, but the functionalities are limited; new programs are hard to develop. At the moment workflow design is bound to existing programs present in the suite, but some workflow builders could be modified to incorporate NGS libraries in the near future.

Visual programming is used in many sectors of software development, such as education, architecture, and video game design. The visual programming philosophy linked to generic template libraries, seen as a powerful extension of workflow builders, can be a valuable aid for improving NGS tool and workflow development. VisPro is a conceptual visual programming framework covering a number of requisites that make it appropriate for development of NGS software (in the need of scalability, transparency, usability, interoperability), especially if coupled with a powerful generic template library, like SeqAn. However, instantiating a brand new VisPro for NGS may require a tremendous development effort. On the other hand, existing general-purpose suites are supported by a large community of developers, users, and investors; even so, they have flaws and may be stalled by further technological changes.

In conclusion, visual programming could effectively bridge the gap between software developers and users needing cutting-edge software, making NGS data science fully translational. While an *ex novo* development of VP software specific for NGS may be unfeasible, trying to improve the visual programming capabilities of existing software and the interoperability with low-level libraries could be a preferred course of action.
